# An investigation of the adsorption of Congo red dye on two naturally occurring adsorbents Hydroxyapatite and Bentonite: An Experimental Analysis, DFT calculations, and Monte Carlo simulation

**DOI:** 10.1016/j.heliyon.2024.e39884

**Published:** 2024-11-01

**Authors:** Ayoub Grouli, Anas Chraka, Yahya Bachra, M'hammed Elkouali, Samir Chtita, Mohammed Berrada

**Affiliations:** a Laboratory of Analytical and Molecular Chemistry, Faculty of Sciences Ben M'Sik, Hassan II University of Casablanca, P.O. Box 7955, Casablanca, Morocco; b Materials and Interfacial Systems Laboratory, ERESI Team. Department of Chemistry, Faculty of Sciences, Abdelmalek Essaâdi, Tetouan University, Morocco

**Keywords:** Congo red dye, Hydroxyapatite, Bentonite, Molecular dynamics, DFT

## Abstract

Congo Red (CR) dye is classified as a toxic and carcinogenic substance, posing significant health and environmental risks. To address this issue, the adsorption efficiency of CR on natural bentonite and hydroxyapatite (HA) was systematically studied. The adsorbents were successfully characterized by XRD, FTIR, and SEM analysis. Optimization through the Box-Behnken method identified the optimal conditions (pH = 6.5, initial dye concentration = 150 mg/L, and adsorbent mass = 1.5 g/L), resulting in maximum removal of CR of 95 % for HA and 84 % for bentonite. 2.6.2. Monte Carlo (MC) simulations provided insights into the spontaneous and favorable adsorption behavior, particularly under acidic conditions, driven by van der Waals interactions. Kinetic studies revealed that the adsorption followed a pseudo-second-order model (R^2^ = 0.99). Furthermore, regeneration tests demonstrated that HA and bentonite retained 75 % and 60 % of their adsorption capacities, respectively, after five cycles, indicating their potential for sustainable reuse in dye removal. The exceptional adsorption efficiency and reusability of these natural adsorbents make them promising candidates for environmental remediation, contributing to a deeper understanding of the underlying adsorption mechanisms.

## Highlights

• Optimized Congo Red adsorption on hydroxyapatite (HA) and bentonite using Box-Behnken Design (BBD).
• Achieved maximum removal efficiencies: 95% for HA and 84% for bentonite under optimal conditions (pH = 6.5, 150 mg/L CR, 1.5 g/L adsorbent).
• Applied Density Functional Theory (DFT) and Molecular Dynamics (MD) simulations to analyze adsorption mechanisms.
• Identified the pseudo-second-order kinetic model as best fitting, with R² = 0.99.
• Confirmed reusability of HA and bentonite, retaining 75% and 60% of adsorption capacity after five regeneration cycles.

## 1 Introduction

Currently, the critical issues of water scarcity, pollution, and quality are at the forefront of global environmental concerns. The rapid pace of technological advancement has introduced new challenges, including increased contamination and environmental disruptions. In this context, the widespread use of dyes across various industrial sectors, particularly in the textile industry, poses significant risks due to their inherent toxicity. Specifically, colored pigments are harmful to both human health and the environment, as many are mutagenic and should not be present in water bodies or effluent streams [1]. Synthetic dyes are extensively employed in various industries such as textiles, printing, rubber, paper, plastics, pharmaceuticals, leather, and cosmetics [2]. Most organic dyes feature complex aromatic structures [3]. Textile effluents are heavily colored due to unfixed dyes and must be treated before disposal. Approximately 30 % of dye production may be wasted during the process [4]. In addition, The discharge of untreated effluent into the environment exacerbates these challenges, posing serious threats to both human health and the global ecosystem [[5], [6], [7]]. Colored effluent, which includes hazardous chemicals such as acids, alkalis, and various toxic-colored contaminants, can disturb catalytic functions. Causing harm to both the environment and human beings [[8], [9], [10]]. Similarly, Congo Red (CR) is a toxic diazo dye characterized by a symmetrical aromatic structure. It exhibits remarkable resistance to photodegradation and biodegradation, along with notable thermal, physicochemical, and optical properties. CR is widely used in industries such as rubber, plastics, paper, and textiles. In water, it initially forms a red colloidal solution before breaking down into benzidine, a known carcinogen to humans and a mutagen to aquatic organisms [[11], [12], [13], [14], [15], [16], [17], [18]]. In response to these environmental challenges, wastewater treatment has garnered global attention, necessitating urgent intervention by scientists to design and optimize efficient methods. A range of physical, chemical, and biological treatment processes are employed to remove dyes from water and wastewater [[19], [20], [21], [22], [23], [24]]. These methods include coagulation, photocatalysis, reverse osmosis, oxidation, ion exchange, adsorption, and ozone treatment. Although most of these methods effectively address the issue of colored water, their main drawbacks are their lack of environmental friendliness and the high costs associated with their operation. Hence, there is a need for pollution removal methods that are both cost-effective and environmentally friendly [[25], [26], [27]]. Adsorption technology is highly effective in removing both organic and inorganic contaminants from water and wastewater. It is favored for its ease of use, cost-effectiveness, high efficiency, and lack of undesirable byproducts, making it a practical wastewater treatment method [28]. Additionally, adsorption technology is straightforward to develop and implement, enabling the removal of various pollutants from water systems. Nonetheless, the challenges of limited selectivity and the generation of hazardous waste due to the difficulty in separating the adsorbent from the pollutants are notable drawbacks of this technique [[29], [30], [31], [32], [33]]. Nanomaterials have recently attracted significant attention in wastewater treatment due to their exceptional properties, such as high adsorption capacity, small particle size, large surface area, high catalytic activity, and thermal stability. A variety of unconventional adsorbents have been investigated, including bentonite, coir pith carbon, fly ash, activated red mud, rice hull ash, leaf, Ag-doped HA, egg shell-based materials, and rice husk. These materials have been used to treat CR from aqueous solutions. While synthetic or modified adsorbents like Ag-doped HA have shown enhanced performance in removing dyes, this study highlights the efficacy of natural adsorbents, offering a sustainable and cost-effective alternative for real-world applications [34]. For the purpose of developing wastewater treatment methods, hydroxyapatite (HA) and bentonite as adsorbents for dye removal must be compared. Because of its large porosity and surface area, HA is an excellent material for creating strong chemical interactions with pollutants, which makes it an excellent choice for eliminating organic and heavy metal contamination [35]. With its layered structure and high capacity for cation exchange, bentonite provides a flexible method for adsorbing a variety of pollutants via surface adsorption, intercalation, and ion exchange [36]. Through the comparison of different materials, the study offers a thorough grasp of their individual advantages and disadvantages, This aids in the selection of the most effective adsorbent for the removal of CR. To bridge this research gap, our study employs cutting-edge Monte Carlo (MC) simulations and density functional theory (DFT) to delve into the nuanced adsorption dynamics of CR on the surfaces of bentonite and HA [[37], [38], [39], [40], [41], [42]].

Leveraging rapid advancements in computer technology, Monte Carlo (MC) simulations have become a powerful tool for addressing the computational and thermodynamic complexities of materials. They offer detailed insights into the behavior of molecular systems and provide comprehensive assessments of interactions within solutions, as well as between adsorbates and adsorbent matrices. Additionally, MC simulations deliver valuable structural details and insights into surface energetics [[43], [44], [45]]. The integration of Density Functional Theory (DFT), often in tandem with MC, enriches our understanding of the intricate interplay between the adsorbate, adsorbent, and polymers, providing a comprehensive view of the adsorption process at the molecular level. In this context, polymers can serve as adsorbents, adding another dimension to the complexity of molecular interactions in adsorption phenomena [[46], [47], [48]].

Our comprehensive study combines experimental analysis with advanced DFT and MC simulations, providing insights into the complexities and mechanisms of CR adsorption using naturally occurring adsorbents. We particularly focus on HA and bentonite surfaces under varying pH conditions. The characterization of HA and bentonite involves sophisticated techniques such as Fourier transform infrared spectroscopy (FTIR), scanning electron microscopy (SEM), and X-ray diffraction (XRD) [43,49,50].

Batch experiments were conducted to assess how varying the adsorbent dosage, pH levels in the aqueous solutions, and initial dye concentration influenced the adsorption process. Employing the powerful statistical tool of Response Surface Methodology (RSM), we optimize operational conditions, dissecting their impact on the targeted response to discern optimal parameters. This holistic approach, integrating experimental insights with computational prowess, enriches our comprehension of CR adsorption behavior, contributing significantly to the evolving landscape of high-impact research in water purification methodologies [[51], [52], [53]].

## 2 Materials and methods

### 2.1 Adsorbents preparation

In the preparation of adsorbents for this study, both HA and bentonite were selected without the need for chemical activation. The native bentonite, sourced from the northern Moroccan region, was sieved to achieve a uniform particle size of 63 μm. Similarly, HA was also sieved to maintain a particle size of 63 μm. One method of synthesizing HA ${Ca}_{10}{\left(PO_{4}\right)}_{6}$ (OH)_2_ involves using hen eggshell waste as a starting material. Hen eggshells are known for their high calcium carbonate content, comprising approximately 95–97 % calcium carbonate. In this process, the eggshells were boiled in hydrogen peroxide to remove organic matter, dried at 100 °C for 12 h, and ground to a particle size of less than 63 μm.

For HA synthesis, the precipitation method was employed. Calcium acetate, derived from eggshells, was prepared by adding excess acetic acid to an eggshell suspension in water, heating, and stirring until a transparent solution formed. After centrifugation, the solution was boiled to evaporate the solvent, yielding a dried product for HA synthesis. The phosphate mineral processing involved a series of steps: drying, grinding, and sieving to separate the phosphate component from the silica component, resulting in particles approximately 63 μm in size. This separation is crucial because silica can interfere with the formation of pure HA, affecting its structural and functional properties. For the one-step chemical precipitation, 10 g of phosphate (63 μm) and 500 ml of deionized water softened with concentrated nitric acid (HNO_3_) to pH = 2 were mixed. The mixture was stirred continuously at ambient temperature for 2 h.

The solution, containing high concentrations of calcium and phosphate ions, was retrieved using filtration. The pH was adjusted to 10 by adding a highly concentrated ammoniacal solution to ensure efficient ultrasonic dispersal of the particles. The resulting precipitated compound was aged for 24 h, followed by filtration and washing with distilled water. The HA was then dried overnight at 100 °C. Calcination was performed at 600 °C for 4 h to complete the preparation process. In the subsequent experimental phases, CR, characterized by the general formula C_32_H_22_N_6_Na_2_O_6_S_2_, was utilized as the model dye to explore its adsorption behavior on bentonite and HA surfaces. Additionally, the sodium bentonite used in this study is derived from the weathering of volcanic ash, which is known for its high cation exchange capacity and ability to absorb large quantities of water. Regeneration experiments employed NaOH (0.1 M) as the desorption agent. All chemicals used were of analytical grade, and distilled water was used in all preparations (Table 1).

**Table 1:** Sample information.

**Compound**	**CAS No**	**Source**	**Initial mass fraction purity**	**Molecular weight (g. mol**^**−**^^**1**^**)**
**Congo Red**	573-58-0	Sigma-Aldrich	35 %	696.66
**HCl**	7647-01-0	Sigma-Aldrich	37 %	36.458
**NaOH**	1310-73-2	Sigma-Aldrich	99 %	39.997
**KNO**_**3**_	7757-79-1	Sigma-Aldrich	99 %	101.103

### 2.2 Adsorption tests

A stock solution of CR dye was prepared for the adsorption tests. Batch mode assays were conducted by adding HA and bentonite to the CR solution at ambient temperature with continuous stirring. CR concentration was measured using a Shimadzu UV–visible spectrophotometer at 497 nm.

The purpose of these experiments was to investigate the adsorption capacity and efficiency of HA and bentonite as a natural adsorbent for CR dye. By measuring the initial and final concentrations of CR in the solution, the removal percentage of the dye by both adsorbents can be determined.

Previous research has extensively investigated the adsorption of CR using bentonite as an adsorbent, employing a variety of techniques to explore its adsorption mechanism and efficiency. Equilibrium experiments have been conducted to determine the maximum adsorption capacity of bentonite for CR. Kinetic analysis has been employed to study the rate at which the adsorption process occurs, providing insights into the adsorption kinetics and the underlying mechanisms. Additionally, spectroscopic studies have been conducted to elucidate the interaction between CR molecules and the surface of bentonite particles, shedding light on the nature of the adsorption process. These studies collectively contribute to a deeper understanding of the potential of HA and bentonite as an effective adsorbent for the removal of CR from aqueous solutions.

The percentage removal of CR in each solution was determined by comparing the initial concentration (C₀) with the equilibrium concentration (C). This calculation was performed using the formula:

(1) $$RC\;\mathrm{Removal}\;\left(\%\right)=\frac{\left({C\;}_{0}-C\right)}{C0}\times 100$$

### 2.3 The point of zero charge (PZC)

The point of zero charge (pH_ZPC_) for both HA and bentonite was experimentally determined using potentiometric titration to assess the surface charge at varying pH levels. A quantity of 0.01 g of each adsorbent (HA and bentonite) was evenly distributed among several 100 mL beakers, each containing 50 mL of an electrolytic medium with a KNO₃ concentration of 0.03 M. The initial pH values (pH_i_) of the solutions were adjusted to range from pH 2 to pH 10. After 24 h of equilibration, the final pH values (pH_f_) were measured. The point at which the pH_i_ equals pH_f_ was identified as the pH_ZPC_ for the adsorbents.

### 2.4 Characterization methods

Fourier transform infrared spectroscopy (FTIR) spectra of HA and bentonite were obtained using KBr pellets on a Bruker Tensor-27 spectrophotometer from Bruker Corporation (Germany). The infrared transmittance method was employed, and all spectra were averaged over 32 scans from 4000 to 400 cm⁻^1^ at a resolution of 4 cm⁻^1^. The X-ray diffraction pattern (XRD) of HA was analyzed using a Bruker D8-Advance X-ray powder diffractometer (Germany) with nickel-filtered Cu-Kα radiation (λ = 1.54056 Å), operating at 40 kV and 100 mA. The diffused radiation was detected over an angle range of 10–80° (2θ), with a stepping point of 0.01° (2θ). Morphological analysis of HA and bentonite was performed using scanning electron microscopy (SEM) with a MiniSEM Hirox model SH-4000M.

### 2.5 Design of adsorption experiments

The experimental setup for the adsorption tests utilized a Box-Behnken design (BBD) to optimize the impact of factors at various stages, including pH (3, 6, 9), CR solution concentration (100, 150, 200 mg/L), and mass of adsorbent (0.5, 1, 1.5 g/L). Experimental data was analyzed and results were extracted using Design-Expert software (version 13.0.5.0).

To evaluate the adsorption efficiency of HA and bentonite, batch tests were conducted according BBD matrix, as shown in Table 2. The procedure involved adding predetermined quantities of the two adsorbents (0.5–1.5 g/L) to 50 mL CR solutions with varying concentrations (100–200 mg/L) and pH values (3–9). pH adjustments were made using 0.5 M solutions of sodium hydroxide (NaOH) and sulfuric acid (H_2_SO_4_).

**Table 2:** BBD matrix and results for both responses.

**Assays**	**pH**	**[CR] (mg/L)**	**Adsorbent mass (g/L)**	**CR removal by HA) (%)**	**CR removal by Bentonite (%)**
**1**	3	100	1	60	51
**2**	9	100	1	69	59
**3**	3	200	1	51	43
**4**	9	200	1	67	56
**5**	3	150	0.5	57	48
**6**	9	150	0.5	60	52
**7**	3	150	1.5	66	57
**8**	9	150	1.5	85	71
**9**	6	100	0.5	82	77
**10**	6	200	0.5	72	63
**11**	6	100	1.5	95	84
**12**	6	200	1.5	87	80
**13**	6	150	1	80	71
**14**	6	150	1	81	70
**15**	6	150	1	80	71
**16**	6	150	1	80	71
**17**	6	150	1	80	71

The number of experimental trials (N = 15) in the BBD matrix was determined using the standard formula (Eq. (2)).

Table 2 summarizes the experimental setup for the adsorption of CR using HA and bentonite. The variables include the pH level, initial CR concentration, and the adsorbent mass. The resulting CR removal percentages for both HA and bentonite are presented as Y_1_ and Y_2_, respectively.

(2) $$N{=k}^{2}+k+C_{p}$$

In this context, the variable k denotes the number of factors, while ${\;C}_{p}$ signifies the number of replicated central points. The BBD resulted in a quadratic regression model (Eq. (3)) linking significant variables (X_1_, X_2_, X_3_) to responses (Y_1_: CR removal by HA (%) and Y_2_: CR removal by bentonite (%)).

(3) $$Y=b_{0}+b_{1}X_{1}+b_{2}X_{2}+b_{3}X_{3}+b_{11}X_{1}^{2}+b_{22}X_{2}^{2}+b_{33}X_{3}^{2}+b_{12}X_{1}X_{2}+b_{13}X_{1}X_{3}+b_{23}X_{2}X_{3}+\varepsilon$$

In a regression model, the predicted response Y depends on three independent variables $X_{1}$, $X_{2}$, and $X_{3}$. The model includes an intercept term $b_{0}$, coefficients for the linear predictors $b_{1},b_{2}$ and $b_{3}$, coefficients for the quadratic predictors $b_{11}$, $b_{22}$ and $b_{33}$, and coefficients for the cross-product predictors $b_{21}$, $b_{13}$, and $b_{23}$ representing interactions between the independent variables.

### 2.6 Computational details

#### 2.6.1 DFT calculations

In addition to the wide array of experimental methods used to investigate CR and its associated properties, this study also complemented the research with theoretical calculations. Utilizing the Density Functional Theory (DFT) methodology and the Gaussian 09 package, we employed the three-parameter Becke, Lee-Yang-Parr (B3-LYP) functional in conjunction with the 6-31G+(d, p) basis sets to optimize the ground-state molecular structure of CR in a gaseous phase [54].

These theoretical calculations extended our understanding of CR by examining optimized parameters related to its electronic properties. This encompassed a detailed exploration of geometric parameters, as well as the characterization of frontier molecular orbitals such as E_HOMO_ and E_LUMO_. Furthermore, we global chemical reactivity descriptors obtained through DFT, including chemical hardness, energy, electronic chemical potential, and electrophilicity, were computed for the molecules under investigation [[55], [56], [57]]. These descriptors play a pivotal role in predicting the relative stability and reactivity of the studied compounds. By assessing these properties, we can gain valuable insights into the behavior and potential reactivity of the title molecules.

By extracting energy values from the highest occupied molecular orbital (HOMO) and lowest unoccupied molecular orbital (LUMO), we were able to compute several essential global chemical descriptors. These descriptors included the energy gap (ΔE = E_LUMO_ - E_HOMO_), ionization energy (I = -E_HOMO_), electronic affinity (A = -E_LUMO_), electronegativity (χ = (I + A)/2), chemical potential μ = −(I + A)/2, and hardness (η = (I - A)/2). These calculations provided valuable insights into the electronic properties of CR and its potential interactions, further enhancing our understanding of this compound and its applications [[58], [59], [60]].

#### 2.6.2 Monte Carlo (MC) simulation

**Fig. 1:**
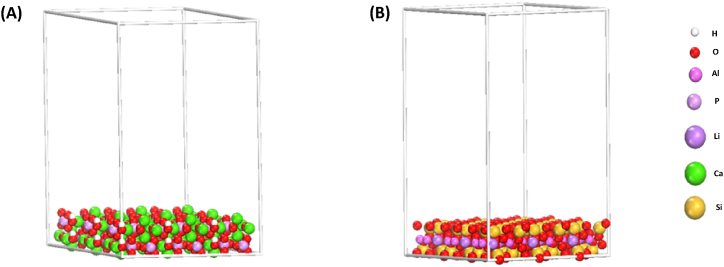
Side images of the (A) HA (001) and (B) MMT (001) surfaces.

In this work, Monte Carlo (MC) simulation was used to investigate the interaction of the CR dye on two naturally occurring adsorbents: HA and bentonite. First, Table 3 shows the HA lattice parameters calculated using first-principles computations. The HA crystal's unit cell parameters are a = b = 9.40 and c = 6.86. A number of modeling studies were conducted to investigate the (001) surface, which was found as the major surface of the HA element. Consequently, we concentrate our attention in this investigation on the interactions of (CR) dye with HA (001) surface (Fig. 1 **(A)).** Since one of the main ingredients of Bentonite is Montmorillonite (MMT), we utilized the MMT (001) surface for the simulated adsorption system in this investigation (Fig. 1(B)), It was selected because of its stability and consistency.

**Table 3:** Calculated lattice parameters of HA and MMT.

**Mode**	**Space group**	**a (Å)**	**b (Å)**	**c (Å)**	**α (°)**	**β (°)**	**γ (°)**	**V (Å**^**3**^**)**
HA	*P*6_3_/*m*	9.40	9.40	6.86	90.00	90.00	120.00	524.90
MMT	C 1 2/m 1	5.17	8.95	9.74	90.00	96.10	90.00	448.57

Table 3 displays the lattice properties of MMT. The lattice parameters of MMT that were determined were a = 5.17, b = 8.95 Å and c = 9.74 Å. The BIOVIA Materials Studio program was used to carry out the MC simulations. To imitate the experimental setting (NaOH media), MC simulation was performed with 500 water (H_2_O), 5 hydroxyls (OH^−^), and 5 sodium (Na^+^) ions, as well as one CR molecule. Furthermore, every simulation box had a thickness of 40 Å vacuum layer at the C axis, corresponding to 3 × 3 × 1 unit cell in each simulation box (28 × 28 × 40 Å). During MC simulations, the potential energy values necessary for molecular interactions were calculated using the COMPASS force field. Moreover, five cycles of simulated annealing at 298 K (20000 steps each cycle) were used in the MC calculations. With a cutoff distance of 12.5, the electrostatic and van der Waals parameters were adjusted to the Ewald summation technique and the atom-based summing approach.

## 3 Results and discussion

### 3.1 Characterization results

**Fig. 2:**
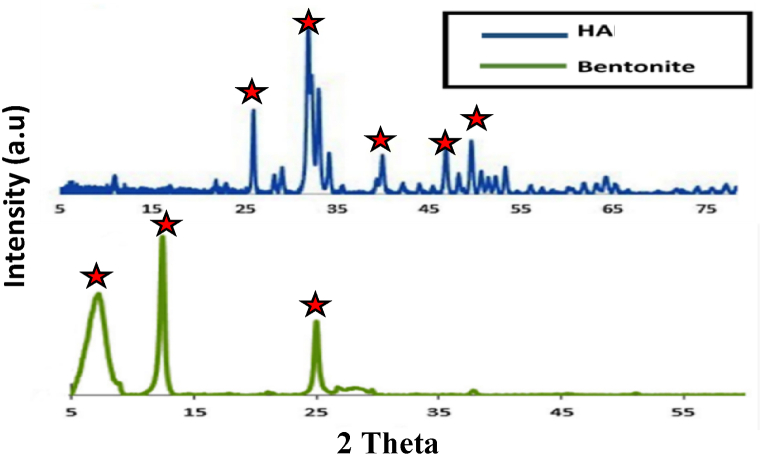
XRD patterns for Bentonite and HA materials.

The X-ray diffraction patterns of HA and bentonite, depicted in Fig. 2, reveal a highly crystalline pure phase for HA, as indicated by distinct peaks at 2θ ≈ 25.9° (002), 31.8° (211), 32.08° (112), 34.03° (202), 36.01° (301), 46.01° (222), and 63.06° (502) (JCPDS 01-086-1199). This suggests that the HA sample is well-defined and suitable for adsorption studies.

The XRD analysis of bentonite, revealed montmorillonite as the primary mineral, which is an aluminum phyllosilicate clay absorbent. Additionally, minor phases of other types of bentonites clay, such as illite and quartz, are also present. The presence of illite in the bentonite sample suggests that it contains this clay mineral, which can impact its adsorption properties and interactions with CR dye.

**Fig. 3:**
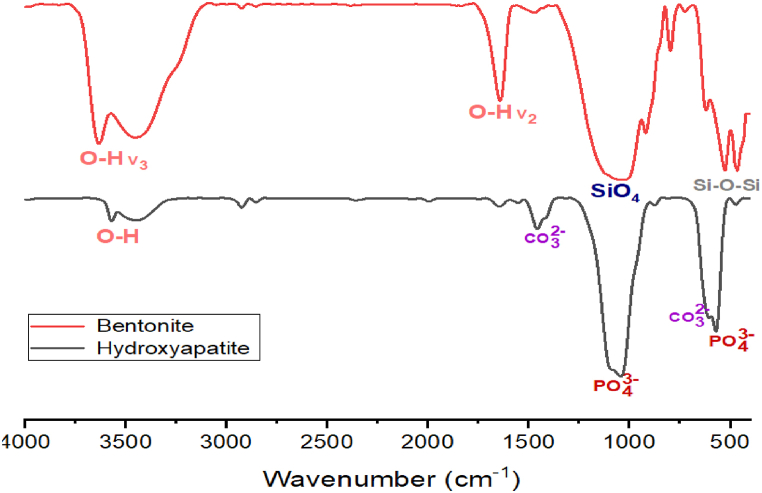
FTIR spectra of HA and Bentonite.

FTIR analysis of bentonite is illustrated in Fig. 3. The spectrum reveals various functional groups and bonds characteristic of the clay mineral composition. Key absorption bands are observed at specific wavenumbers: around 3620 cm⁻^1^ (O-H stretching), 3420 cm⁻^1^ (H-O-H stretching of adsorbed water), 1640 cm⁻^1^ (H-O-H bending of water molecules), 1040 cm⁻^1^ (Si-O stretching), 790 cm⁻^1^ (Si-O-Si bending), and 520 cm⁻^1^ (Al-O-Si bending). This analysis helps identify the specific components and their interactions, providing insights into the structural and chemical properties of the bentonite sample.

The transmission FTIR spectra of HA show a band between 3300 and 3550 ${cm}^{-1}$ attributed to the vibrational mode of hydroxyl groups, and bands located at 1455, 1420, and 875 ${cm}^{-1}$ indicate the presence of ${CO}_{3}^{2-}$. The bands at 1030, 605, 564, and 468 ${cm}^{-1}$ correspond to the ${PO}_{4}^{3-}$.

**Fig. 4:**
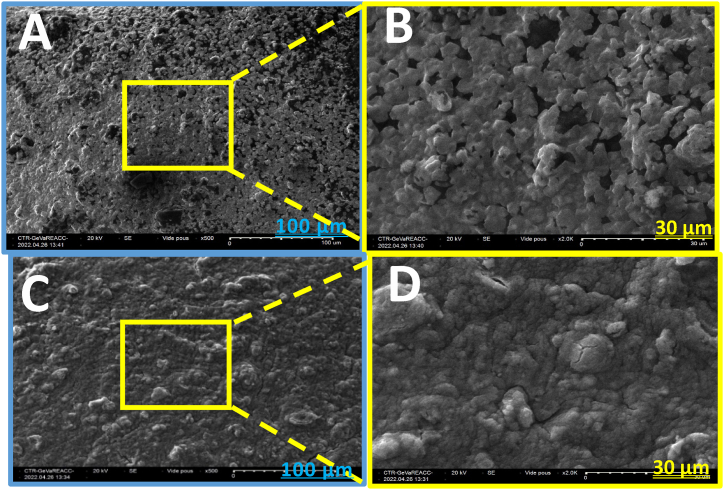
SEM images: (A) and (B) HA; (C) and (D) Bentonite.

The SEM images of hydroxyapatite (HA) and bentonite (Fig. 4) reveal significant differences in their surface morphology and porosity, which are likely to influence their adsorption behavior. HA exhibits a highly irregular surface with particles of varying shapes and sizes, creating interparticle voids that enhance its overall porosity. This irregularity suggests a higher surface area, which could provide more active sites for adsorption, contributing to its effectiveness in removing contaminants like Congo Red. In contrast, bentonite displays a more homogeneous, mesh-like structure, indicating a network of interconnected pores. This structure may facilitate rapid diffusion of Congo Red molecules throughout the material, allowing for efficient adsorption in the initial stages. However, the lower surface roughness of bentonite suggests fewer active sites than HA. These structural differences highlight that while HA may offer greater adsorption capacity due to its extensive surface area, bentonite's interconnected porosity promotes faster adsorption kinetics.

### 3.2 pH of zero-point charge

**Fig. 5:**
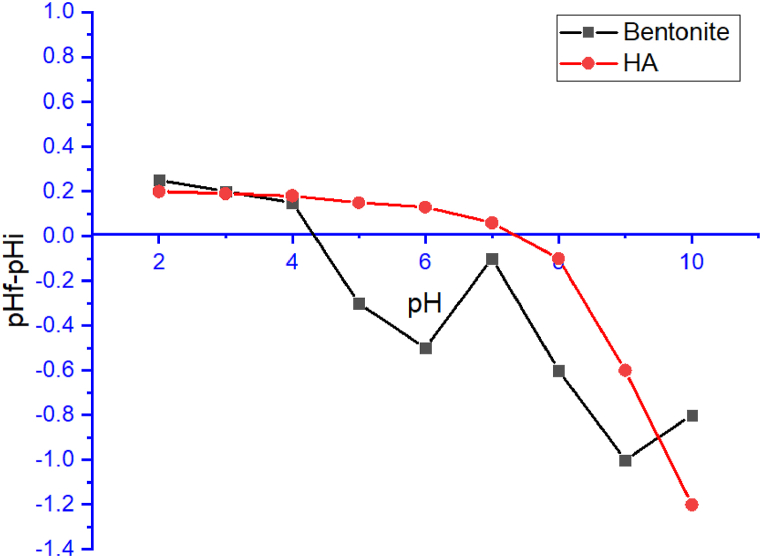
pHzpc of HA and bentonite.

The pH of the solution significantly influences the adsorption capacity of hydroxyapatite (HA) and bentonite due to the variation in their surface charge at different pH levels (Fig. 5). The pH at the zero point of charge pH_zpc_ for HA is approximately 6.2, which means that at pH values below 6.2, the surface of HA is positively charged, facilitating the adsorption of the negatively charged CR molecules through strong electrostatic interactions. As the pH increases beyond the pH_zpc_, the surface charge of HA becomes negative, leading to a reduction in electrostatic attraction, although adsorption can still occur via van der Waals forces and hydrogen bonding. Similarly, bentonite, with a pH_zpc_ around 4, exhibits a strong negative surface charge at pH levels higher than 4, favoring the adsorption of anionic CR through cation exchange and surface adsorption. In acidic conditions, bentonite's surface becomes less negatively charged, slightly reducing its adsorption capacity. The optimal pH for maximum adsorption, observed around 6.5 for both adsorbents, results from the balance between electrostatic attraction and other interaction forces, confirming that pH control is critical in maximizing dye removal efficiency.

### 3.3 Adsorption study

#### 3.3.1 Optimization of the CR adsorption using BBD

In this study, the adsorption of Congo Red (CR) on hydroxyapatite (HA) and bentonite was evaluated using response surface methodology (RSM) and analysis of variance (ANOVA). The validity of the RSM model was assessed through fit statistics and ANOVA results, including the coefficient of determination ($R^{2}$), adjusted $R^{2}$, and predicted $R^{2}$. In principle, an $R^{2}$ greater than 0.8 and a p-value less than 0.05 indicate model validity (Table 4), agreement with experimental measurements, and the significance of factor coefficients. Accordingly, higher $R^{2}$ values (close to 1) and lower p-values (<0.0001) support the validity of both models and confirm a strong correlation between experimental and predicted responses.

**Table 4:** Adjustment statistics for both responses.

$R^{2}$	**Response 1**	**Response 2**
	0.997	0.997
**Adjusted**$R^{2}$	0.994	0.992
**Predicted**$R^{2}$	0.968	0.944
**Adeq precision**	63.267	52.199
**Std. Dev**	0.977	1.05
**Mean**	74.24	64.41
**Coefficient of Variation (%)**	1.32	1.63

The ANOVA analysis (Table 5) revealed that the interactions between pH and [CR], as well as between pH and adsorbent mass, are significant factors. Additionally, the quadratic effects of pH and adsorbent mass were significant with p-values below 0.05 for all four terms. The adsorption of CR was significantly influenced by the pH of the solution, CR concentration, and adsorbent mass. Increasing the pH from 6 to 8 and adsorbent mass from 0.5 to 1.5 g/L resulted in a substantial improvement in CR adsorption, with maximum removal efficiencies of 95 % for HA and 84 % for bentonite observed at pH values between 6 and 7, and adsorbent mass of 1.3–1.5 g/L. However, a clear decrease in performance was noted when the pH exceeded 7.

**Table 5:** ANOVA values for the quadratic pattern of responses Y1 and Y2.

	**Response 1**		**Response 2**	
	F−value	p−value	F−value	p−value
Model	304.18	<0.0001	220.02	<0.0001
A−pH	276.79	<0.0001	163.03	<0.0001
B−[Congored]	151.22	<0.0001	108.50	<0.0001
C−Adsorbentmass	502.83	<0.0001	317.15	<0.0001
AB	12.82	0.0072	8.13	0.0246
AC	75.61	<0.0001	32.52	0.0007
BC	0.26	0.6228	18.29	0.0037
$B^{2}$	1377.67	<0.0001	1249.36	<0.0001
$C^{2}$	65.22	<0.0001	1.49	0.2624

The combined effect of pH and CR concentration on the adsorption process indicated that as the pH increased from 3 to 7, the removal efficiency of CR improved gradually, regardless of the initial concentration. Nevertheless, at higher CR concentrations (100–200 mg/L), a slight decrease in adsorption efficiency was observed, particularly for bentonite. This suggests that while higher pH values generally enhance adsorption capacity, the efficiency diminishes slightly at higher dye concentrations, likely due to the saturation of available adsorption sites on bentonite.

**Fig. 6:**
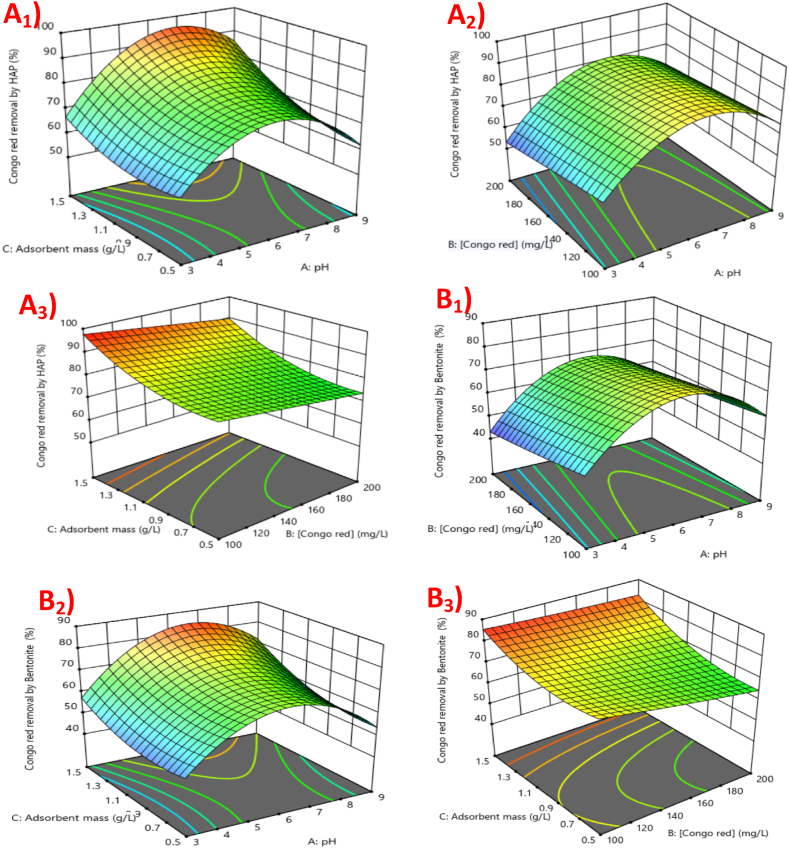
3D response surface plots of CR removal by HA (A_1_, A_2_, A_3_) and bentonite (B_1_, B_2_, B_3_) show the effect of adsorbent mass and pH at [CR] = 100 mg/L.

The 3D response surface plots (Fig. 6) illustrate the combined effects of pH, Congo Red concentration, and adsorbent mass on CR removal efficiency for both hydroxyapatite (HA) and bentonite. The plots reveal that the adsorption efficiency improves with increasing pH, reaching a maximum at pH 6–7, particularly when the adsorbent mass is between 1.3 and 1.5 g/L. For HA, CR removal efficiency is highest (95 %) under optimal conditions, while bentonite shows slightly lower removal (85 %) under similar conditions. The efficiency decreases at higher Congo Red concentrations (>150 mg/L) for both adsorbents, likely due to the saturation of available adsorption sites. These trends emphasize that adsorbent mass and pH are critical parameters for optimizing CR removal, while higher dye concentrations can limit adsorption capacity.

In conclusion, the optimum conditions were determined using Design-Expert 13 software and are summarized in Table 6. Under optimal conditions of pH = 6.5, [CR] = 150 mg/L, and adsorbent mass = 1.5 g/L, the CR removal efficiencies were 95 % for HA and 85 % for bentonite. The predicted performances were verified by experimental tests, confirming that both materials are effective adsorbents for high concentrations of CR.

**Table 6:** Optimum conditions for CR removal.

**pH**	**6.5**	
**[CR] (mg/L)**	150	
**Adsorbent mass (g/L)**	1.5	
**CR Removal Efficiency by HA (%)**	**Predicted**	97
	**Experimental**	95
**CR Removal Efficiency by Bentonite (%)**	**Predicted**	85
	**Experimental**	84

**Fig. 7:**
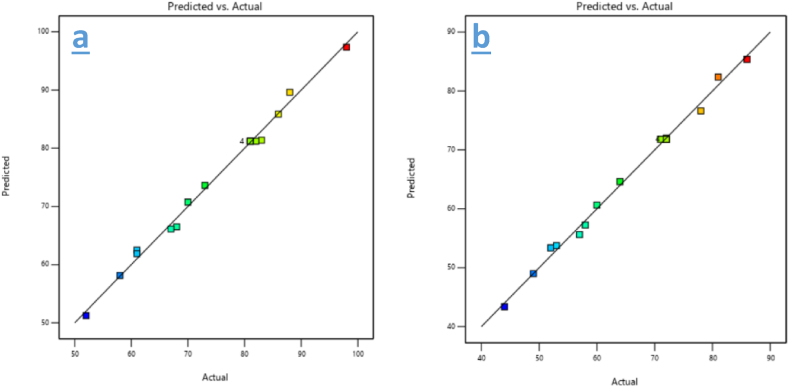
Predicted vs Actual graphs of CR removal by: a) HA, b) Bentonite.

The predicted vs. actual graphs for CR removal by HA and bentonite in Fig. 7 demonstrate the accuracy of the applied models in predicting removal efficiency. In the case of HA (a), the points closely follow the line of perfect correlation, indicating a strong agreement between the predicted and actual values, reflecting the model's high reliability for CR adsorption. For bentonite (b), the graph also shows a good correlation, though there is slightly more deviation compared to HA, suggesting that while the model remains reliable, its predictive accuracy for bentonite may be somewhat lower. Overall, these graphs validate the model's effectiveness, confirming that both HA and bentonite are efficient adsorbents for CR removal under the tested conditions. The slight deviations observed for bentonite may point to a more complex interaction between variables that affects its adsorption behavior.

### 3.4 Kinetic modeling of CR adsorption onto HA and bentonite adsorbents

To provide a comprehensive understanding of the adsorption kinetics, both the pseudo-first-order and pseudo-second-order kinetic models were applied to the experimental data for the adsorption of Congo Red onto hydroxyapatite (HA) and bentonite.

The pseudo-first-order model is expressed by Equation (4), which assumes that the rate of adsorption is proportional to the number of available sites:

(4) $$\ln\left(q_{e}-q_{t}\right)=\ln\left(q_{e}\right)-k_{1}t$$

• $q_{e}$ (mg.g⁻^1^), is the amount of adsorbate at equilibrium,
• $q_{t}$ (mg.g⁻^1^), is the amount of adsorbate at time t
• $k_{1}$ (mg.g⁻^1^.min⁻^1^) is the pseudo-first-order rate constant,
• t (min) is the contact time.

Based on the data in Table 7, the pseudo-first-order model did not fit the experimental data well, especially for hydroxyapatite (HA). For example, at 50 mg/L, the $R^{2}$ value was only 0.615, and the calculated ${\;q}_{e}$ value (8.870 mg/g) was significantly lower than the experimental $q_{e}$ value (23.62 mg/g), indicating a poor fit.

**Table 7:** First-order and pseudo-second-order model parameters.

**Adsorbent**			**Pseudo first order**			**Pseudo second order**		
	**Concentration**	$q_{e}\text{,}$**experimental (mg.g**^**−**^**^1^)**	$k_{1}$**(**$\min^{-1}$**)**	$q_{e}\text{,}$**calculated (mg.g**^**−**^**^1^)**	$R^{2}$	$k_{2}$ $\left(g.{\text{mg}}^{-1}.\min^{-1}\right)$	$q_{e}\text{,}$**calculated (mg.g**^**−**^**^1^)**	$R^{2}$
**HA**	10	5.03	0.068	1.603	0.952	0.378	5.147	0.99817
	30	14.57	0.106	6.525	0.674	0.296	15.788	0.986
	50	23.62	0.077	8.870	0.615	0.156	25.329	0.985
	100	40.01	0.059	2.582	0.707	0.012	40.209	0.999
**Bentonite**	10	3.20	0.020	3.001	0.986	0.094	3.132	0,998
	30	3.50	0.003	3.482	0.995	0.009	3.871	0.972
	50	10.32	0.005	10.071	0.984	0.002	10.482	0.997
	100	24.74	0.002	23.833	0.968	0.002	25.80	0.994

In contrast, the **pseudo-second-order model**, described by Equation (5), assumes that chemisorption is the rate-limiting step, and provides a much better fit to the experimental data:

(5) $$\frac{t}{q_{t}}=\frac{1}{k_{2}q_{e}^{2}}+\frac{t}{q_{e}}$$

In Eq. (5), $k_{2}$ is the pseudo-second-order rate constant (mg.g⁻^1^.min⁻^1^), $q_{e}$ is the amount of adsorbate at equilibrium (mg.g⁻^1^), $q_{t}$ is the amount of adsorbate at time t (mg.g⁻^1^), and t is the contact time (min).

For HA, the pseudo-second-order model provided significantly better fitting, with $R^{2}$ values approaching 0.999. For instance, at 100 mg/L, the experimental $q_{e}$ value (40.01 mg/g) closely matched the calculated $q_{e}$ value (40.209 mg/g), indicating a strong agreement between the model and the experimental data.

Similarly, for bentonite, the pseudo-second-order model provided a better fit than the pseudo-first-order model. For example, at 100 mg/L, the $R^{2}$ value for the pseudo-second-order model was 0.994, compared to 0.968 for the pseudo-first-order model. The calculated ${\;q}_{e}$ value of 25.80 mg/g was also closer to the experimental $q_{e}$ value of 24.74 mg/g, further supporting the accuracy of the pseudo-second-order model.

The higher rate constants ($k_{2}$) for both adsorbents indicate a faster adsorption rate, particularly for HA. This, along with the higher $R^{2}$ values, suggests that chemisorption is the dominant mechanism in the adsorption of Congo Red onto both HA and bentonite.

In conclusion, the results show that the pseudo-second-order model is more suitable for describing the adsorption of Congo Red onto HA and bentonite, highlighting the role of chemisorption as the rate-limiting step in the process.

### 3.5 DFT results

#### 3.5.1 Optimized structure

**Fig. 8:**
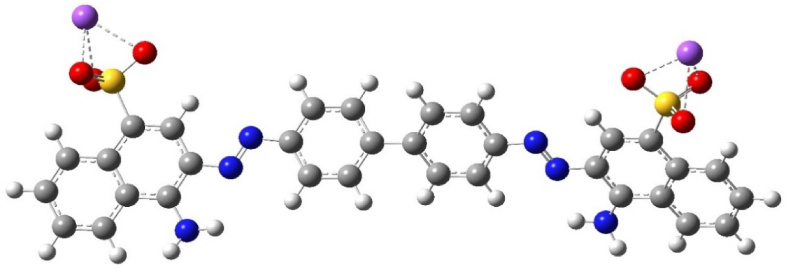
Optimized structure of the CR at the B 3LYP/6-31 + G(d,p) level of DFT calculations in gas phase.

The utilization of the DFT has unquestionably proven to be immensely advantageous when it comes to elucidating the intricate details of molecular structures, electronic properties, and the reactivity of molecules. Fig. 8 illustrates the optimized molecular structure of the CR molecule.

#### 3.5.2 Frontier molecular orbitals

**Fig. 9:**
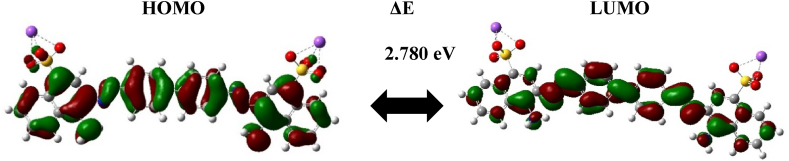
The contour plots of FMO for the investigated compound (CR).

Fig. 9 provides visual representations of the frontier molecular orbitals (FMO), specifically the Highest Occupied Molecular Orbital (HOMO) and Lowest Unoccupied Molecular Orbital (LUMO) for the CR. Through the analysis of the HOMO and LUMO, we gain insight into the electron density distribution within the optimized molecular structures. Fig. 9 illustrates that both the LUMO and HOMO exhibit electron densities that are evenly dispersed across the entire molecule. These orbitals display strong delocalization within the conjugated system. It's important to note that the contour plots of the HOMO and LUMO are structure-dependent, highlighting that the electron density of the HOMO is predominantly concentrated on atoms with a delocalized character. This observation implies that these particular atoms serve as favored sites for adsorption within the compound being studied. This detailed analysis enhances our comprehension of the molecule's electronic characteristics, shedding light on its reactivity and behavior.

#### 3.5.3 Global reactivity descriptors

The optimization and analysis of the HOMO-LUMO energy gap, along with other descriptors, provide valuable insights. In Table 8 presents the simulated values of the energies of the frontier molecular orbitals (FMOs) and global chemical reactivity descriptors for CR at the DFT/B3LYP/6–31G (d, p) level of theory.

**Table 8:** Optimization energies, HOMO and LUMO energies and their gap calculated in gas phase at B3LYP/6-31 + G(d, p) level of DFT calculations.

**Descriptors**	**eV**
**E**_**HOMO**_	−5.084
**E**_**LUMO**_	−2.304
**ΔE**_**gap**_	2.780
**I**	5.084
**A**	2.304
**χ**	3.694
**μ**	−3.694
**η**	1.390

The E_HOMO_ and E_LUMO_ values shed light on the donor-acceptor interaction between the CR dye onto the surface of the studied materials. An increase in the E_HOMO_ value becoming less negative and the E_LUMO_ value becoming more negative indicates improved electron donation and acceptance capabilities from the studied materials. From the data in Table 8, the HOMO orbital has an energy of −5.084 eV, and the LUMO orbital has an energy of −2.304 eV. The higher energy is attributed to electron-donating groups, specifically heteroatoms like oxygen and nitrogen.

The energy band gap is a key determinant of the electronic and optical proprieties of the system under study. A larger energy gap (ΔE) suggests that the molecule is less likely to interact and adsorb onto a surface. This is because a wider energy gap indicates a greater difference between the highest occupied molecular orbital (HOMO) and the lowest unoccupied molecular orbital (LUMO) of the CR molecule. A larger gap signifies weaker electronic interactions and electron transfer with the adsorbent surface, resulting in less favorable adsorption behavior. As indicated in Table 8, the calculated band energy gap of CR equals 2.780 eV implies stronger adsorption interactions and increased adsorption capacity for the CR molecule on the studied surface.

The hardness pertains to the resistance of an adsorbent to adsorb or bind specific molecules. It relates to the selectivity and specificity of the adsorption process. A “hard” surface is less likely to interact with molecules possessing particular electronic or steric requirements, while a “soft” surface can accommodate a wider range of molecules due to its flexible nature. A high hardness value indicates the surface's strong preference for adsorbing specific molecules, suggesting selectivity in its adsorption behavior. When hardness is high, the adsorbent can accommodate only a limited range of adsorbates and favors interactions with those that meet specific electronic or steric criteria. In Table 8, the calculated hardness of CR, equal to 1.390 eV, indicates a moderate preference for adsorbing specific molecules.

Electronegativity, in the context of adsorption, refers to the relative ability of molecules to attract and retain electrons when they interact with a surface material. Higher electronegativity values in molecules lead to polar interactions with the surface, resulting in stronger adsorption, while lower electronegativity may lead to weaker adsorption interactions. As for the 3.694 eV hardness value in Table 8, this high value suggests that CR has a strong ability to attract electrons within chemical bonds, indicating a greater tendency for polar or ionic interactions with the adsorbent surface, which can lead to stronger adsorption interactions.

### 3.6 MC modeling results

**Fig. 10:**
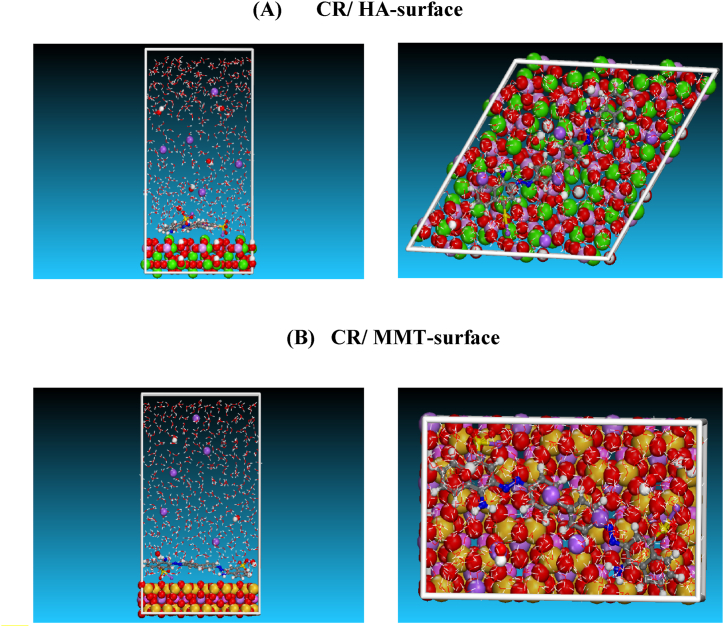
The final snapshots of the most stable low-energy configuration for the adsorption retrieved from MC for: (A). CR/HA-surface; (B). CR/MMT-surface.

For a better understanding of the relevance and possibility of occurrence of adsorption interactions. Recognizing the adsorption energy (E_ads_), which is the sum of the deformation energy (E_RA_) and deformation energy (E_Def_) of the additive elements, is critical. Furthermore, the Eads can aid in the classification of the adsorbate's constituent parts. As in the case of the current investigation, the experimental outcomes can thus be anticipated or validated (Fig. 10). The different types of energetic descriptors i.e., total energy (E_Total_), E_ads_, E_RA_, E_Def_, dE_ads_/dNi_CR_, dE_ads_/dNiH_2_O dE_ads_/dNiOH^−^ and dE_ads_/dNiNa^+^ of adsorbed CR in the aqueous phase on HA-surface and MMT-surface are computed and presented in Table 9. It is important to note that persistent or simpler adsorption onto the surface is indicated by larger and negative values of E_ads_ [20,21]. Furthermore, Table 9 makes it evident that all of the E_ads_ values for the CR/HA-surface and CR/MMT-surface systems are negative, indicating that the adsorption process that results from the surface contacts of the adsorbents HA (001) and MMT (001) with the CR molecule is spontaneous. Additionally, the following values were ascertained by the examination of adsorption energy: Compared to the CR/HA-surface system, which is closer to −4764.40 kcal/mol, the CR/MMT-surface system is closer to −4503.57 kcal/mol. The adsorption energy (E_ads_) results indicating that the HA surface has a lower adsorption energy compared to the MMT surface in a basic solution suggest that HA has a weaker interaction with the adsorbate than MMT. This means that the adsorbate binds more strongly to the MMT surface in basic conditions. This information could be critical in determining which adsorbent is more effective under specific pH conditions for your study on the adsorption of CR dye.

**Table 9:** Outputs and descriptors for the: CR/HA-surface; CR/MMT-surface systems during MC simulations at 298 K (all values in Kcal/mol).

**System**	**E**_**tot**_	**E**_**ads**_	**E**_**RA**_	**E**_**Def**_	**dE**_**ads**_**/dN**_**CR**_	**dE**_**ads**_**/dNi**_**H2O**_	**dE**_**ads**_**/dNi**_**OH**_^**-**^	**dE**_**ads**_**/dNi**_**Na**_^**+ -**^
CR/HA-surface	−3665.54	−4764.40	−4154.14	−610.26	−165.55	−11.81	−2.46	−0.28
CR/MMT-surface	−3404.71	−4503.57	−3880.67	−622.90	−150.51	−10.21	−2.50	−0.53

**Fig. 11:**
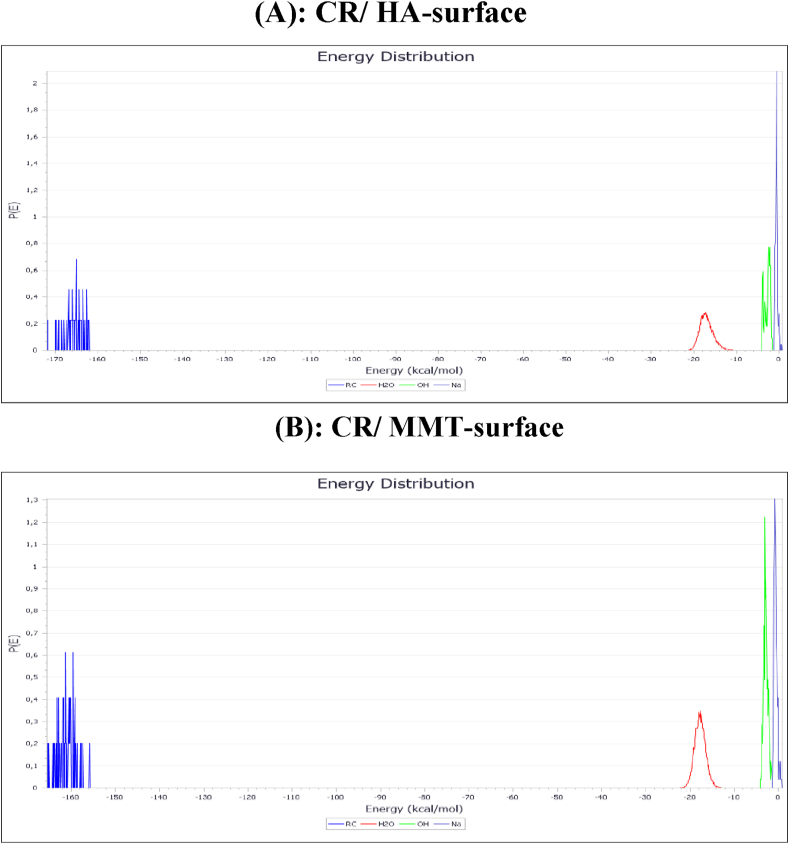
Adsorption energy distribution for the: (A). CR/HA-surface; (B). CR/MMT-surface systems during MC simulations.

Fig. 11 depicts the adsorption energy distribution for CR/HA-surface and CR/MMT-surface systems during MC simulations. As indicated in Fig. 11(A and B), the adsorption energy of CR on HA-surface and MMT-surface is (−172 Kcal/mole) and (−165 Kcal/mole), respectively.CR adsorption is stronger on two clays, with a preference for HA with a high ability to absorb CR.

### 3.7 Regeneration test results

**Fig. 12:**
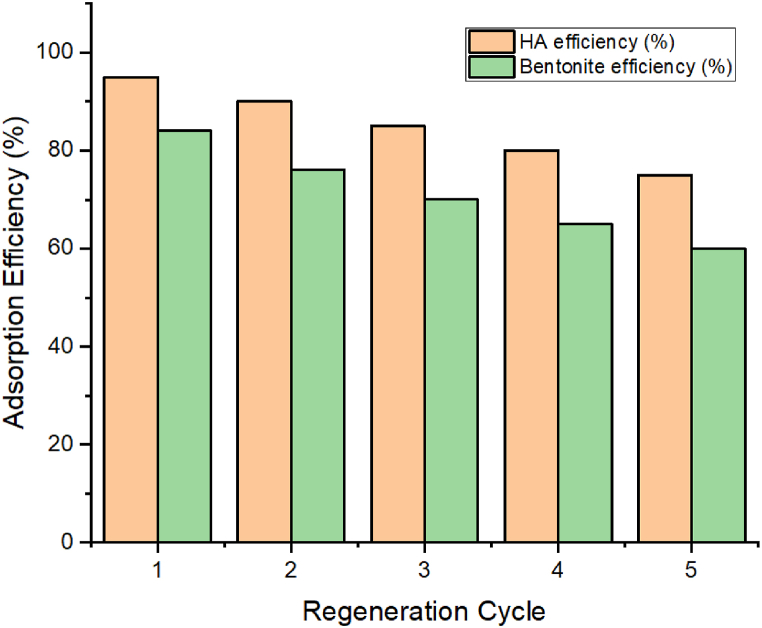
Regeneration test for HA and bentonite.

The results of the regeneration test (Fig. 12) indicate that both hydroxyapatite (HA) and bentonite maintain significant adsorption efficiency over several regeneration cycles. Initially, HA exhibited a 95 % removal efficiency, which decreased to 75 % after five cycles. Bentonite showed slightly lower initial efficiency at 84 %, dropping to 60 % after the same number of cycles. This decrease in performance can be attributed to the partial saturation of adsorption sites and possible structural changes in the adsorbents during the regeneration process. Nevertheless, both materials retained sufficient adsorption capacity, indicating their potential for reuse in industrial applications. HA, with its higher retention of adsorption efficiency, proved to be more durable than bentonite under repeated use. The consistent performance of HA can be linked to its greater structural integrity and higher surface area, which allows for more efficient regeneration and reuse.

## 4 Conclusion

This study investigated the adsorption of CR dye from aqueous solutions using HA and bentonite as natural, inorganic adsorbents. Adsorption conditions were optimized using BBD model, revealing strong agreement between experimental and predicted data for variables such as pH, adsorbent mass, and CR concentration. Under optimal conditions (pH = 6.5, [CR] = 150 mg/L, and adsorbent mass = 1.5 g/L), HA and bentonite exhibited maximum CR removal efficiencies of 95 % and 84 %, respectively. Further, quantum chemistry approaches, including Density Functional Theory (DFT) calculations and Monte Carlo (MC), provided insights into the physicochemical stability and adsorption mechanisms of CR on these materials. Both simulations and experimental data confirmed that CR adsorbed onto HA and bentonite in a parallel orientation, with significant negative adsorption energies indicating spontaneous adsorption. van der Waals interactions dominated the adsorption process, and kinetic studies showed that the pseudo-second-order model accurately described the adsorption behavior.

Moreover, regeneration tests demonstrated that HA and bentonite retained 75 % and 60 % of their initial adsorption capacities, respectively, after five cycles of reuse. This highlights the reusability and sustainability of these materials for repeated adsorption applications, making them promising candidates for scalable wastewater treatment processes.

The study offers a comprehensive understanding of the adsorption mechanisms of CR on HA and bentonite, underscoring the potential of these natural adsorbents in environmental remediation. The incorporation of quantum chemical modeling, kinetic analysis, and regeneration performance provides a robust framework for future research. Continued exploration of the adsorbent-adsorbate interface and optimization of regeneration techniques will be critical to advancing adsorption technologies and improving the efficiency of pollutant removal in industrial wastewater treatment.

Future work should focus on enhancing the long-term efficiency and sustainability of hydroxyapatite and bentonite as adsorbents. This can be achieved by exploring surface modifications to further improve their adsorption capacities for a wider range of pollutants, including heavy metals and other industrial dyes. Additionally, scaling up these materials for use in continuous flow systems will be crucial for real-world applications. Investigating the regeneration processes in greater detail, particularly over extended cycles, could provide further insights into their economic viability. Finally, expanding the use of computational methods, such as advanced molecular dynamics simulations, could help predict adsorption behaviors under various environmental conditions, allowing for the development of more targeted and efficient water treatment strategies.

## CRediT authorship contribution statement

**Ayoub Grouli:** Writing – review & editing, Writing – original draft, Formal analysis, Data curation, Conceptualization. **Anas Chraka:** Writing – review & editing, Writing – original draft, Formal analysis, Data curation, Conceptualization. **Yahya Bachra:** Visualization, Validation, Conceptualization. **M'hammed Elkouali:** Visualization, Validation. **Samir Chtita:** Visualization, Validation, Project administration, Investigation. **Mohammed Berrada:** Visualization, Validation, Supervision, Project administration.

## Ethics approval and consent to participate

The study does not require ethical approval. The study did not involve any animal or human data or tissue.

## Consent for publication

All authors have read and agreed to the published version of the manuscript.

## Data availability

The datasets generated and/or analyzed during the current study are available from the corresponding author on reasonable request.

## Funding

The authors did not receive support from any organization for the submitted work.

## Declaration of competing interest

The authors declare that they have no known competing financial interests or personal relationships that could have appeared to influence the work reported in this paper.
